# Older adults’ perceptions and experiences of interprofessional communication as part of the delivery of integrated care in the primary healthcare sector: a meta-ethnography of qualitative studies

**DOI:** 10.1186/s12877-024-04745-4

**Published:** 2024-02-12

**Authors:** Karoline Stray, Torunn Wibe, Jonas Debesay, Asta Bye

**Affiliations:** 1 Department of Nursing and Health Promotion, Faculty of Health Sciences, OsloMet - Oslo Metropolitan University, Oslo, Norway; 2 Centre for Development of Institutional and Home Care Services, City of Oslo, Oslo, Norway; 3 Department of Nursing and Health Promotion, Faculty of Health Sciences, OsloMet - Oslo Metropolitan University, Oslo, Norway; 4 Department of Nursing and Health Promotion, Faculty of Health Sciences, OsloMet - Oslo Metropolitan University, Oslo, Norway; 5 European Palliative Care Research Centre (PRC), Department of Oncology, Oslo University Hospital, and Institute of Clinical Medicine, University of Oslo, Oslo, Norway

**Keywords:** Interprofessional communication, Primary health care, Older adults, Meta-ethnography, Integrated care

## Abstract

**Background:**

Communication between patients and healthcare providers, and effective interprofessional communication, are essential to the provision of high-quality care. Implementing a patient-centred approach may lead to patients experiencing a sense of comfort, validation, and active participation in own healthcare. However, home-dwelling older adults’ perspectives on interprofessional communication (IPC) are lacking. The aim is therefore to explore how home-dwelling older adults experience communication in connection with the delivery of integrated care.

**Methods:**

The meta-synthesis was conducted in line with Noblit and Hare’s seven phases of meta-ethnography. A systematic literature search was conducted by two university librarians in seven databases using the search terms ‘older adults’, ‘communication’, ‘integrated care’ and ‘primary care’. All articles were reviewed by two authors independently. 11 studies were included for analysis.

**Results:**

Older adults are aware of IPC and have preferences regarding how it is conducted. Three main themes were identified in the reciprocal analysis: (1) Inconsistent care perceived as lack of IPC, (2) individual preferences regarding involvement and awareness of IPC and (3) lack of IPC may trigger negative feelings.

**Conclusions:**

This meta-ethnography shows the perspective of older adults on IPC as part of integrated care. Our study shows that older adults are concerned about whether healthcare personnel talk to each other or not and recognise IPC as fundamental in providing consistent care. The perspectives of older adults are relevant for clinicians and politicians, as well as researchers, when developing and implementing future integrated care services for home-dwelling older adults.

**Supplementary Information:**

The online version contains supplementary material available at 10.1186/s12877-024-04745-4.

## Introduction

Health and social services are recommended to deliver integrated care, especially when attending to older adults’ declining intrinsic capacity [1, 2]. A broad definition of integrated care is ‘an organising principle for care delivery that aims to improve patient care and experience through improved coordination’ [3]. This method of organizing complex care processes is expected to reduce possible fragmentation of patient services, and to increase coordination and continuity [3]. In addition, it is reported that community-living older adults receiving integrated care services, perceive improved quality [4, 5] and enhanced satisfaction with care [5].

A review of 15 reviews of integrated care shows that the key elements of integrated care models for older adults involve multidisciplinary care teams, comprehensive assessment and case management [6]. When working in multidisciplinary teams it is necessary to spread knowledge across the workforce which calls for inter-professional communication (IPC), and communication has been highlighted as an important element within integrated care [4]. Moreover, integrated care that involves tailoring treatment underpinned by clear communication strategies, may have positive effects on re-admissions, mortality and functional decline [7], and there is a need for improved understanding of integrated care practices that support communication across health and social care providers [6].

To enable health professionals to provide consistent and relevant information to the patient it is important that the information communicated between key stakeholders in healthcare services is clear and consistent [8, 9]. Low variability in information and care keeps treatment within a narrow range of practice, leading to more efficient and improved outcomes [10]. IPC e.g. at meetings, is necessary to keep health professionals up-to-date about the current status of the patient [11], thereby leading to more consistency. At the same time it is shown that adoption of a personal and person-centred approach, may lead to patients that feel reassured [12], recognised and involved in own healthcare [13]. Despite this acknowledgment of the importance of IPC in primary healthcare services, there is lack of information about how older adults perceive IPC and its necessity. Such information can be important for facilitating IPC and for motivating healthcare personnel to talk together.

### Statement of the problem

Several studies conclude that communication between patients and healthcare providers and effective IPC are essential to the provision of high-quality care [14, 15]. However, a review of home-dwelling older adults’ perspectives on IPC is lacking. The aim of this meta-synthesis of qualitative studies was therefore to elaborate on and explore how home-dwelling older adults experience IPC in connection with the delivery of integrated care. The review question was as follows:

- How do older adults experience IPC when receiving primary healthcare services?

## Method

We conducted a meta-ethnography, a well-known systematic approach that compares texts to arrive at a holistic interpretation [16]. This approach includes seven phases [16],: 1) getting started, 2) deciding what is relevant to the initial interest, 3) reading the studies, 4) determining how the studies are related, 5) translating the studies into one another, 6) synthesising translations and 7) expressing the synthesis. We used the guide ‘Using a meta-ethnography in health care research’ [17] in addition to the eMERGe meta-ethnography reporting guidance from France et al. [18]. The study was registered in PROSPERO with record number CRD42021221900.

### Phase 1 – getting started

The initial stage of the meta-synthesis involved examining the current literature regarding the perspectives of home-dwelling older adults on IPC in primary healthcare settings. Initially, we conducted a search in the Epistemonikos and PROSPERO databases to determine if any relevant systematic reviews on the subject were available. To identify primary studies that were pertinent to our research, the first author performed a structured test search in CINHAL. Additionally, we carried out unstructured searches in Google Scholar. These searches encompassed various areas, including integrated care, communication, older adults, primary care, patient experience, and qualitative/mixed methods.

### Phase 2 – deciding what is relevant

After clarifying the topic of interest, the authors decided on the focus of the synthesis [17]. The literature search was conducted by two librarians, while the search strategy was reviewed by a third librarian. All three librarians were from at the University Library, Oslo Metropolitan, Oslo, Norway. Based on the health perspective of the research questions, the literature review was conducted in six databases: MEDLINE (OVID), EMBASE (OVID), APA PsycINFO (OVID), the Cochrane Central Register of Controlled Trials (CENTRAL), CINAHL (EBSCO), Web of Science and Google Scholar. The search was conducted between April and May 2021. In Medline, Embase, PsycInfo, Cinahl and Web of Science, the authors received weekly updates on articles published between May 2021 and September 2022. We utilized five search components in our study: older adults, experience/perception, integrated care, interprofessional communication/collaboration, and community health care services. We also implemented the snowball method. Synonyms and pertinent terms were identified and combined in the search process. Below are examples of the search terms and keywords associated with each component:

- Older adults: Elderly, Senior*, Geriatric
- Integrated care: Person-centered care, People-centered
- Interprofessional communication/collaboration: Cross-Disciplinary Communication*, Multidisciplinary Communication*, Interdisciplinary Communication*
- Community health care services: Municipal service, Primary health care, Comprehensive health care

The full search history with search terms is available in Additional file 1.

The first author initiated the list of inclusion criteria (Table 1), which was shared with the university librarian. Qualitative and mixed-method studies were included, with the inclusion of mixed-method studies based on the quality and relevance of the qualitative research findings. Articles were initially reviewed based on titles and abstracts, followed by full-text screening using Covidence review management software [19]. Articles with a wide age range were included only if the age demographics were explicitly mentioned in relation to participant quotes and findings. Articles lacking specificity about each participant or affiliated quotes were excluded.

**Table 1:** List of inclusion criteria

Criteria	Inclusion	Exclusion
**Population**	• Home-living older adults (65+)	• Younger adults • Children • Older adults living in nursing homes
**Language**	• English • Scandinavian	• All other languages
**Year limit**	• Unlimited	• September 2022
**Type of publication**	• Journal articles • PhD thesis	• Reviews • Poster abstracts • Editorials
**Study type**	• Qualitative • Mixed Methods	• Quantitative
**Setting**	• Community health care • Primary care	• Hospital • Transitions
**Type of qualitative data**	• Semi-structured • In-depth interviews	
**Phenomenon of interest**	• Interprofessional communication • Information continuity	• Interpersonal communication between healthcare provider and patient

**Fig. 1:**
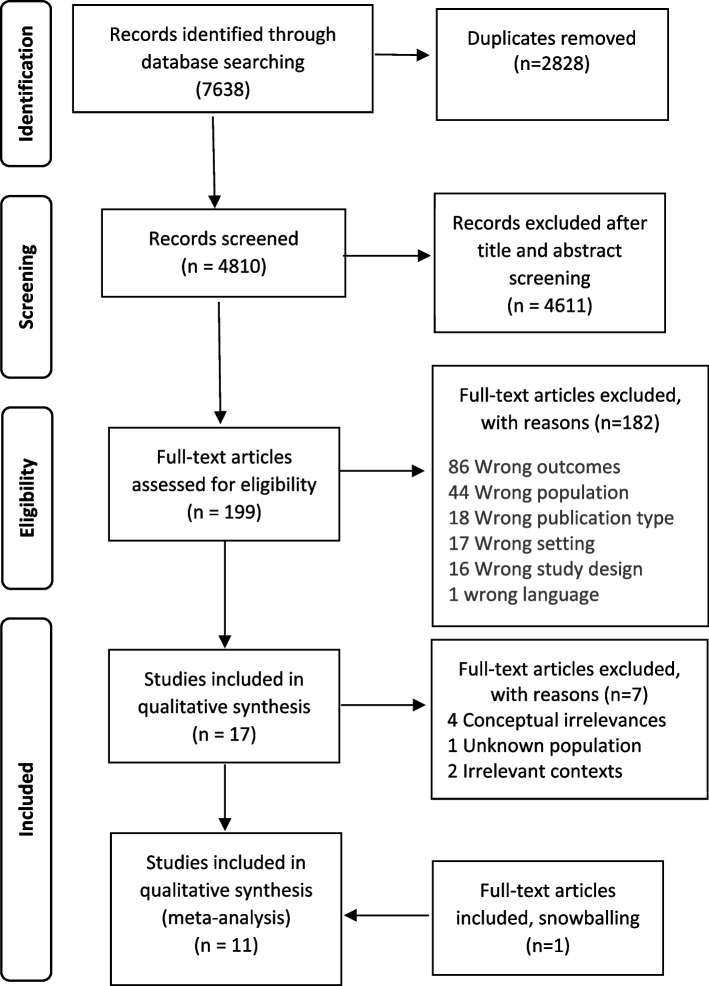
PRISMA diagram, selection of primary studies. From:  Moher D, Liberati A, Tetzlaff J, Altman DG, The PRISMA Group (2009). Preferred Reporting Items for Systematic Reviews and Meta-Analyses: The PRISMA Statement. PLoS Med 6(7): e1000097. doi:10.1371/journal.pmed1000097. For more information, visit  www.prisma-statement.org

Two authors independently reviewed all articles, with conflicts resolved through discussion and agreement. The selection of primary studies is visualized in the PRISMA diagram (Fig. 1).

We used the checklist of the Critical Appraisal Skills Program (CASP, https://casp-uk.net/casp-toolschecklists/) to systematically assess the quality of each article. Every article was assessed by two manuscript authors independently, and disagreements were discussed with the involved authors until agreement was reached. Included studies are presented in Additional file 2.

### Phase 3 – reading the included studies

The first author thoroughly read each article to gain a deep understanding of key concepts and metaphors [16, 17]. Data were extracted using a data extraction table (see Additional file 3) which included first-order constructs (participant quotations), second-order constructs (primary authors’ interpretation of primary data) and associated themes or concepts [17]. The raw data were further summarized in a column labelled ‘what is this about’, reducing/merging first and second-order constructs to a short descriptive explanation. Study characteristics were extracted in a separate table (see Additional file 2). The extraction table was presented at a workshop held on 16 May 2022 and the authors agreed on the relationship between first-order and second-order constructs, and ‘what is this about’. Further, we discussed whether a reciprocal translation or refutational synthesis was most appropriate based on the extraction tables and the study characteristics.

### Phase 4 – determining how studies are related

The studies were regarded as related when they described experiences of primary health care from the perspectives of patients. Articles that primarily involved the perspectives of health professionals and families were considered relevant if they included patients’ experiences. Despite the differences in the aims of primary studies, all the studies identified or explained new concepts related to communication between health professionals. All primary studies took a similar approach to data collection and analysis, but differed in terms of their contexts, such as country and type of healthcare intervention or service (e.g., general practice, end-of-life care etc.). The studies were situated in primary care, except one that concerned both primary care and hospital care [20], however, relevant data could be extracted from the primary care context. The studies are interconnected due to comparable findings, similar population, and contexts.

### Phase 5 – translating the studies into one another

During this phase, a column titled ‘Descriptive labels’ [17] was added to the data extraction table. These labels were developed from first and second-order constructs, summarizing each row under the heading ‘what is this about’. The primary studies were initially organised alphabetically, starting with Aerts et al. [21], which was compared to Borgsteede et al. [22], commenting on similar and contrasting concepts related to the study context [17], before moving to the next study. Since the phases of analysis were overlapping and repeated [16], some primary studies did not follow the alphabetical order. Using ‘descriptive labels’, first-order data and second-order themes were categorised in a separate translation table to explore how the studies could be interconnected. The process allowed for a comparison and alignment of first and second-order constructs [17].

### Phase 6 – synthesising the translation

Through a reciprocal translation, we explored older adults’ views and experiences of interprofessional communication. This phase involved identifying third-order constructs, which are the reviewer’s interpretation of a tertiary analysis of first and second-order constructs. To develop third-order constructs, the first authors moved back and forth between the translation table, the data extraction table, and the primary studies to ensure that the interpretations represented something more than the parts alone imply [16].

### Phase 7 – expressing the synthesis

In this phase, the focus was on enabling researchers and clinicians to identify how older adults notice and are affected by IPC by investigating ‘terms of others’ interpretations and perspectives’ [16]. To be able to express ‘construct-adequate metaphoric translation’, it was relevant to confer with representatives of primary health professionals to ensure that the result of the synthesis is expressed in a way that makes sense to the audience/relevant stakeholders [16].

### Reflexivity

The first author has previously worked as a clinical home care nurse in Norway. She recognized some of the situations described in the primary studies. To mitigate potential bias, the first author reflected on her previous encounters and corresponding preconceptions related to interprofessional communication in home care. Additionally, the second author has experience within primary care, working in the Health Agency within a municipality. It is worth highlighting that three out of the four authors of this study are nurses. This fact underscores the necessity to ensure that our analysis accurately represents the viewpoints of home-dwelling older adults. To counteract this potential bias, the third author’s experience as a qualitative researcher and the last author’s profession as a dietitian have played crucial roles in challenging and refining our interpretation of the findings.

## Results

A total of 199 studies were read in full text and 182 were excluded, mainly due to wrong outcomes (*n* = 86) (not focusing on home-dwelling older adults views on IPC), wrong population type (*n* = 44) (not > 65 or not living at home), and wrong publication type (*n* = 18) (books, research protocols, conference abstracts). Furthermore, four articles were excluded based on conceptual irrelevance [23–26], one [27] due to difficulties extracting first and second-order constructs that, with certainty, represented older adults’ views, and two because the studies were not performed in a relevant context [28, 29]. No articles were excluded based on the quality assessments. All articles were considered to be of good quality (range 7–10) and were equally emphasized in the analyses.

Three main themes were identified through the reciprocal analysis: (1) Inconsistent care perceived as lack of IPC, (2) individual preferences regarding involvement and awareness of IPC and (3) lack of IPC may trigger negative feelings. A summary of interrelationships between data from the primary studies and new interpretations are presented in Table 2.

**Table 2:** Overview of the synthesis of translation and themes

Third order constructs / Main themes	Descriptors (groups of similar concepts developed from first and second order data, clustered together)	Articles contributing to first and/or second-order data
Inconsistent care perceived as lack of IPC	Health professionals’ concordance The importance of a red thread Communication and Quality of care	[20, 21, 30–33, 37]
Individual preferences regarding involvement and awareness	Different reactions to IPC IPC and exclusion of older adults	[22, 31, 33–37]
Lack of IPC may trigger negative feelings	Repeating history and frustrations Inadequate IPC resulting in feelings of insecurity	[20, 21, 31–34, 37]

### Inconsistent care perceived as lack of IPC

In the study by Wells et al. (2020), the older adults stated that they expected health professionals to discuss their case or healthcare plan, and they conveyed a clear expectation that IPC would contribute to better quality of care. This was expressed through statements such as: ‘Collaboration between any providers is always necessary no matter what the subject matter. Proper communication makes for good performance. Improper communication makes for squat’ [30], and ‘When communication was evident among the team members and with the older person and the family, the care was perceived as consistency’ [20].

When health professionals gave divergent advice, or if an older adult noticed that health professionals disagreed about their care plan, this was considered to represent poor IPC and indicated that health professionals were not working together [31]. Conversely, we found that the older adults believed that the health professionals were collaborating when they did not have to repeat themselves [21]. At times, there was inconsistency between what an older adult considered correct information and what the health professionals considered important to communicate. This led the older adults to be ‘alert and keep a sharp watch on the information transfer between professionals and the formal care arrangements themselves, in order to avoid misunderstandings and errors’ [32]. When health professionals had difficulties talking to other health professionals, even though they interacted with the same patient, one older adult argued that this could be because medical practitioners are often ‘medical alpha dogs’ [33]. This can be understood as someone with a dominant appearance, who often perceives their own course of action to be the right one to pursue, without consulting others.

### Individual preferences regarding awareness and involvement in IPC

Some older adults may feel excluded if they discover that health professionals have talked about them without involving them directly, as described in the following: ‘The home service workers and the home healthcare nurses have a weekly meeting, but we clients are never informed about what they discuss’ [34]. Not being directly involved could lead to the clients feeling ‘treated like passive bystanders in their own care process and that the professionals make decisions for them instead of with them’ [35].

While some older adults felt excluded as a result of not being involved in the IPC, others stated that having a health professional acting as a liaison felt supportive and that they did not need to be directly involved [36]. One person said: ‘She [the GP] takes everything quietly. She talks to the nurses about those pills: Should we do this, or should we do that? Well, that’s [what] it’s all about, isn’t it?’ [22]. The older adults in Eloranta et al. [34] did not express a need to be involved when the health professionals discussed their case, and in Lyons et al. [31], one older adult expressed that they would not mind their case being discussed without them taking part.

### Lack of IPC may trigger negative feelings

The primary studies included in this synthesis report that older adults experience frustration due to poor or inefficient IPC. This is attributed to health professionals working in silos with no communication, which can lead to e.g. repetition of assessments [33, 37]. Personal feelings were affected when the older adults experienced a lack of communication. This involved feelings of being devalued, anxiety and a perception of suboptimal care [20]. Some older adults viewed lack of communication as synonymous with lack of concern for them. This was expressed by one older adult who felt that the health professionals had failed to communicate his needs: ‘They don’t give a damn about you’ [20].

## Discussion

This meta-ethnography suggests that IPC does not go unnoticed among older adults receiving primary healthcare services. Older adults perceive IPC as a prerequisite for consistency and quality of care. If they felt that the healthcare personnel gave divergent advice or incorrect follow-up, it was interpreted as a lack of communication between the healthcare personnel. Becoming aware of inadequate IPC seemed to trigger negative feelings such as frustration, anxiety and the perception of suboptimal care. Some of the older adults wanted to be directly involved when healthcare personnel exchanged information about them, or at least to be informed about what was said, while others felt supported despite not being involved.

Interprofessional collaboration can affect the delivery of healthcare, but there is not enough evidence on how interprofessional collaboration interventions work [38]. Primary healthcare providers face ideological, organisational, structural and relational challenges related to collaboration [39], and there is little research on the mechanisms behind the success of collaboration. IPC is an important mechanism to provide interprofessional collaboration, as collaborative interactions are the result of individuals working together and communicating openly [40]. Historically, the organisation of Western healthcare systems has taken a disease-focused approach to patients, where communication was often a one-way dialogue, e.g., doctors told nurses what to do [40, 41]. Paradoxically this disease-focused approach causes fragmentation of services and is a threat to a holistic perspective on primary care [41]. The results of this meta-ethnography show that older adults feel that a lack of IPC is synonymous with inconsistent care, which could be related to this paradox. From the perspective of older adults, it could be the case that primary care still takes a disease-focused approach, where health professionals treat diseases differently in the various levels of specialisations or professions [41], e.g. when health professionals give divergent advice [31]. The paradox occurs when older adults expect to receive integrated primary care services that include consistency in care. However, according to our results, they do not feel that they receive such care when IPC is inadequate. This paradox may explain why older adults feel that having to repeat their history to different members of the healthcare team represents a lack of IPC, as different professions have different perspectives they must address in disease-focused care. One example of this is physiotherapists’ focus on self-management and rehabilitation, in contrast to nurses’ focus on care and medication. As such, when the physiotherapist visits the patient, they might ask the patient to repeat their history to address the physiotherapist’s perspective of self-management, while the nurse asks for the patient’s history to identify e.g. medical errors or side-effects. To the patient, this might be experienced as the same history, but the different professions use the history differently to develop a treatment plan – a physiotherapy plan and nursing plan, respectively. This might mean that primary care remains disease-focused rather than integrated, where IPC is performed in a way that prevents the older adult from having to repeat their story.

Integrated care has its roots in the Bio-Psycho-Social Model, where ‘every system is influenced by its environment’ [42]. Healthcare systems need to acknowledge cultural and existential dimensions within care [43]. This opens for healthcare becoming about more than just diseases, and makes ‘person-focused care’ or ‘person-centred care’ a central tenet of integrated care. Person-focused care is based on personal preferences, needs and values [41], and can be related to how health professionals should empower, facilitate and support older adults to build on their strengths, make their own decisions and manage their own health [44]. Our results emphasise this by reflecting on user involvement and how some older adults want to be involved in IPC. Despite this, however, we see that patient involvement in dialogue and decision-making regarding IPC is often lacking [45]. It can be challenging to involve older adults in team meetings as this requires a shared understanding of what role the older adult is expected to have in the meeting, and it is also time-consuming [46]. In the context of home care services, the health professional interacts with various other health professionals during the day. It is difficult for the older adult to always be involved in the IPC as interactions are daily and often unplanned [47]. To be able to meet the individual perceptions and needs of older adults regarding IPC, we suggest that healthcare services work in line with integrated care, where the goal is ‘to optimise the interface between different sectors and professional groups from the patient’s perspective’ [9].

Despite challenges with involving older adults in IPC, it is not less important. However, our results also show that older adults have individual preferences concerning involvement in IPC, and that not everyone wants to be involved. These findings on individual preferences regarding user involvement are in line with another study, which emphasises the importance of supporting and tailoring the level of involvement [46]. This suggests that involving older adults in IPC is not necessarily more time consuming and nor does it demand greater resources, as it only applies to some older adults. To identify which older adults want to be involved in IPC, health professionals can conduct holistic and comprehensive assessments in order to provide services that are tailored to their preferences [44].

It has been challenging to find other studies that look at IPC from the perspective of older adults. However, we found one meta-summary about continuity with some similar findings to this meta-ethnography. There, patients were found to experience continuity negatively in the form of uncertainty, insecurity, lostness, vulnerability or mistrust [48], while we found that lack of IPC led to frustration, anxiety, devaluation and the perception of suboptimal care. It is important to note that the meta-summary about continuity had defined communication as a dimension of continuity, and continuity is acknowledged as an important outcome of integrated care [3]. Distinguishing between communication, collaboration and communication can be challenging as the terms are often overlapping, with sometimes similar definitions. This could explain why patients and older adults express similar negative feelings regarding continuity and lack of IPC, as it might be difficult for patients to respond to how they feel about these somewhat overlapping concepts. However, based on how their perspectives take on different nuances of negative feelings than those expressed by patients regarding continuity, e.g. feelings of frustration versus feelings of uncertainty, we argue that IPC is different from continuity. Another argument for how IPC differs from continuity and collaboration is that IPC goes beyond the exchange of information, and involves elements of ‘understanding common objects’ [49].

### Strengths and limitations

To the best of our knowledge, this is the first systematic review to look at home-dwelling older adults’ experiences as an important aspect of communication between health professionals in the provision of healthcare services. One strength of this study is that we provide a thorough description of the method. We strived for transparency, and by following known guidelines for reporting meta-ethnography, we have reported in detail what is considered important.

Articles featuring informant samples with a wide age range but lacking specificity about age for participants’ quotes in the result section were excluded. This might introduce potential knowledge bias since there could be other primary articles that would have been included if they had detailed the age demographics in connection with the quotes and results.

The material was mainly descriptive, and since many of the studies involved other populations in addition to older adults, the relevant data we extracted were quite thin. We tried to conduct a lines-of-argument analysis, but as few studies solely focused on older adults’ perspectives, we were not able to conduct thicker descriptions and further interpret the data [50]. One reason for the limited evidence might be the language restrictions we imposed. We have only included studies in English or a Scandinavian language, and all the included studies originate from ‘Western countries’. This could represent a potential bias, as Western countries might have a different perspective on integrated care than non-Western countries.

During phases 3, 4, 5, and 6 of the research, the first author’s prior knowledge and experiences, particularly as a clinical home care nurse, may have had an impact on the interpretation of the findings [51]. Recognizing these experiences and preconceptions has been important in order to avert interpretations that conform solely to the first author’s initial understanding and obstruct the emergence of new interpretations that could lead to new insights. We believe that this has been avoided through awareness of the importance of identifying possible preconceptions and expanding understanding through joint discussions throughout the interpretation process.

## Conclusion

Our main findings were that older adults are concerned about whether healthcare personnel talk to each other or not, and recognise IPC as fundamental to providing consistent care. Assurance that the healthcare personnel had spoken about and been informed of their condition made them feel safe. As regards the older adults’ need to be informed about or directly involved in IPC, we found that preferences varied. Some wanted to be involved or at least know what had been said about them, while others just needed to feel confident that their situation had been conveyed to the various professionals. The perspectives of older adults are relevant for clinicians and politicians, as well as researchers, when developing and implementing future integrated care services for home-dwelling older adults. Further research should investigate how IPC affects the mechanisms of collaboration, as well as provide thick descriptions of the older adults’ experiences and perceptions of IPC.

### Supplementary Information

**Additional file 1. **Search History

**Additional file 2.**

**Additional file 3.**

## Data Availability

Generated data is available in additional files 2 and 3.

## Abbreviations

IPC: Interprofessional communication
G: General practitioner
LBP: Low Back Pain
