# Supplementary material for: Older adults’ perceptions and experiences of interprofessional communication as part of the delivery of integrated care in the primary healthcare sector: a meta-ethnography of qualitative studies
Source: BMC Geriatr. 2024 Feb 12;24:146. doi: 10.1186/s12877-024-04745-4 (PMC10863142; doi:10.1186/s12877-024-04745-4)
Supplement: Supplementary file 1 — Additional file 1. Search History [file 12877_2024_4745_MOESM1_ESM.docx]

# **Additional file 1. Search History**

**Database:** Ovid MEDLINE(R) and Epub Ahead of Print, In-Process, In-Data-Review & Other Non-Indexed Citations and Daily <1946 to April 28, 2021>

**Date:** 29.04.2021

**Hit number:** 1953

| **#** | **Searches** | **Results** |
| --- | --- | --- |
| 1 | "Aged, 80 and over"/ or Aged/ or Health Services for the Aged/ or Frail Elderly/ or Geriatrics/ | 3253990 |
| 2 | Focus groups/ or Interviews as topic/ or Patient satisfaction/ or Qualitative research/ or Patient Preference/ or Narration/ | 225674 |
| 3 | (qualitative or phenomenolog* or hermeneu*).tw,kw,kf. | 276701 |
| 4 | 2 or 3 | 435245 |
| 5 | 1 and 4 | 82665 |
| 6 | ((aged or elder* or eldest or old* or geriatric* or senior* or aging or ageing) adj4 (experience* or perception* or perspective* or view* or opinion* or interview* or findings or preference* or focus group* or satisf*)).tw,kw,kf. | 46146 |
| 7 | 5 or 6 | 123920 |
| 8 | case management/ or "delivery of health care, integrated"/ or "continuity of patient care"/ or patient-centered care/ | 60683 |
| 9 | ((integrated or person cent* or personcent* or patient cent* or patientcent* or patient focus* or patientfocus* or person focus* or personfocus* or seamless or continuity or continuum) adj3 (healthcare or care or service*)).tw,kw,kf. | 44626 |
| 10 | 8 or 9 | 90954 |
| 11 | Patient Care Team/ or Interprofessional Relations/ or Interdisciplinary Communication/ or Intersectoral collaboration/ or Physician-nurse relations/ | 128170 |
| 12 | Communication Barriers/ or Communication/ or Interdisciplinary Communication/ or Cooperative Behavior/ or Health Information Exchange/ or Intersectoral collaboration/ | 148089 |
| 13 | 11 and 12 | 35140 |
| 14 | ((communicat* or inform* or coordinat* or cooperat* or collaborat* or misinform* or miscommunicat* or mis inform* or mis communicat*) adj4 (interprofession* or inter profession* or interdisciplin* or inter disciplin* or multidisciplin* or multi disciplin* or cross disciplin* or crossdisciplin* or care team* or intersector* or inter sector*)).tw,kw,kf. | 19366 |
| 15 | 13 or 14 | 50931 |
| 16 | 10 or 15 | 137209 |
| 17 | community health centers/ or community mental health centers/ or community health services/ or community health nursing/ or home health nursing/ or parish nursing/ or community mental health services/ or community pharmacy services/ or home care services/ or home nursing/ or senior centers/ or rural health services/ or rural nursing/ or suburban health services/ or Nursing Homes/ or Respite care/ or Homebound Persons/ or Homes for the Aged/ or Assisted living facilities/ | 171827 |
| 18 | ((community or municipal* or home or homes or primary or local*) adj3 (service* or health* or care)).tw,kw,kf. | 299157 |
| 19 | 17 or 18 | 419077 |
| 20 | 7 and 16 and 19 | 1953 |

**Database:** **Ovid Embase**1974 to 2021 April 28

**Date:**  29.04.2021

**Hit number:** 2813

| **#** | **Searches** | **Results** |
| --- | --- | --- |
| 1 | aged/ or frail elderly/ or very elderly/ or geriatrics/ | 3184065 |
| 2 | interview/ or semi structured interview/ or structured interview/ or telephone interview/ or unstructured interview/ or patient satisfaction/ or patient preference/ or qualitative research/ or narrative/ | 510918 |
| 3 | (qualitative or phenomenolog* or hermeneu*).tw,kw. | 346069 |
| 4 | 2 or 3 | 736725 |
| 5 | 1 and 4 | 111130 |
| 6 | ((aged or elder* or eldest or old* or geriatric* or senior* or aging or ageing) adj4 (experience* or perception* or perspective* or view* or opinion* or interview* or findings or preference* or focus group* or satisf*)).tw,kw. | 58357 |
| 7 | 5 or 6 | 162796 |
| 8 | integrated health care system/ or patient care/ or case management/ | 334102 |
| 9 | ((integrated or person cent* or personcent* or patient cent* or patientcent* or patient focus* or patientfocus* or person focus* or personfocus* or seamless or continuity or continuum) adj3 (healthcare or care or service*)).tw,kw. | 62074 |
| 10 | 8 or 9 | 371177 |
| 11 | interdisciplinary communication/ or interpersonal communication/ or communication barrier/ or cooperation/ or intersectoral collaboration/ or doctor nurse relation/ | 222075 |
| 12 | intersectoral collaboration/ or multidisciplinary team/ or collaborative care team/ or interdisciplinary communication/ | 27318 |
| 13 | ((communicat* or inform* or coordinat* or cooperat* or collaborat* or misinform* or miscommunicat*) adj4 (interprofession* or inter profession* or interdisciplin* or inter disciplin* or multidisciplin* or multi disciplin* or cross disciplin* or crossdisciplin* or intersector* or inter sector* or care team*)).tw,kw. | 27609 |
| 14 | 11 and 12 | 15328 |
| 15 | 13 or 14 | 40866 |
| 16 | 10 or 15 | 403224 |
| 17 | community care/ or community based rehabilitation/ or community health nursing/ or community mental health service/ or senior center/ or home care/ or nursing home/ or respite care/ or homebound patient/ or home for the aged/ or assisted living facility/ | 194867 |
| 18 | ((community or municipal* or home or homes or primary or local*) adj3 (service* or health* or care)).tw,kw. | 392183 |
| 19 | 17 or 18 | 527834 |
| 20 | 7 and 16 and 19 | 2813 |

**Database:**  **APA PsycInfo**1806 to April Week 3 2021
**Date:** 29.04.2021

**Hit number:** 408

| **#** | **Searches** | **Results** |
| --- | --- | --- |
| 1 | geriatric patients/ or geriatrics/ or gerontology/ or Aging/ or Older Adulthood/ or ("380" or "390").ag. | 368622 |
| 2 | qualitative methods/ or exp focus group/ or semi-structured interview/ or exp interviews/ or phenomenology/ or client satisfaction/ or Narratives/ | 66327 |
| 3 | (qualitative or phenomenolog* or hermeneu*).tw. | 218103 |
| 4 | 2 or 3 | 255987 |
| 5 | 1 and 4 | 23757 |
| 6 | ((aged or elder* or eldest or old* or geriatric* or senior* or aging or ageing) adj4 (experience* or perception* or perspective* or view* or opinion* or interview* or findings or preference* or focus group* or satisf*)).tw. | 38838 |
| 7 | 5 or 6 | 59342 |
| 8 | integrated services/ or patient centered care/ or case management/ or exp "continuum of Care"/ | 9355 |
| 9 | ((integrated or person cent* or personcent* or patient cent* or patientcent* or patient focus* or patientfocus* or person focus* or personfocus* or seamless or continuity or continuum) adj3 (healthcare or care or service*)).tw. | 15633 |
| 10 | 8 or 9 | 21372 |
| 11 | Interdisciplinary Treatment Approach/ | 7424 |
| 12 | Collaboration/ or Cooperation/ or Communication Barriers/ or Communication/ or Interpersonal Communication/ | 68542 |
| 13 | ((communicat* or inform* or coordinat* or cooperat* or collaborat* or misinform* or miscommunicat*) adj4 (interprofession* or inter profession* or interdisciplin* or inter disciplin* or multidisciplin* or multi disciplin* or cross disciplin* or crossdisciplin*)).tw. | 6330 |
| 14 | 11 and 12 | 575 |
| 15 | 13 or 14 | 6640 |
| 16 | 10 or 15 | 27439 |
| 17 | community services/ or social services/ or community mental health services/ or community welfare services/ or home care/ or home visiting programs/ or public health services/ or community health/ or nursing home residents/ or nursing homes/ or Public Health Service Nurses/ or Respite Care/ or homebound/ or Residential Care Institutions/ | 67315 |
| 18 | ((community or municipal* or home or homes or primary or local*) adj3 (service* or health* or care)).tw. | 101347 |
| 19 | 17 or 18 | 144454 |
| 20 | 7 and 16 and 19 | 408 |

**Database:** **Cinahl with full text** (EBSCO)

**Dato:** 30.04.2021

**Antall treff:** 1666

| **#** | **Query** | **Results** |
| --- | --- | --- |
| S1 | (MH "Frail Elderly") OR (MH "Aged, 80 and Over") OR (MH "Aged") OR (MH "Geriatrics") OR (MH "Health Services for the Aged") | 866,859 |
| S2 | (MH "Focus Groups") OR (MH "Interviews+") OR (MH "Patient Preference") OR (MH "Patient Satisfaction") OR (MH "Qualitative Studies+") OR (MH "Narratives") | 364,646 |
| S3 | TI ( (qualitative or phenomenolog* or hermeneu*) ) OR AB ( (qualitative or phenomenolog* or hermeneu*) ) | 147,641 |
| S4 | S2 OR S3 | 410,976 |
| S5 | S1 AND S4 | 80,554 |
| S6 | TI ( (aged or elder* or eldest or old* or geriatric* or senior* or aging or ageing) n3 (experience* or perception* or perspective* or view* or opinion* or interview* or findings or preference* or "focus group*" or satisf*) ) OR AB ( (aged or elder* or eldest or old* or geriatric* or senior* or aging or ageing) n3 (experience* or perception* or perspective* or view* or opinion* or interview* or findings or preference* or "focus group*" or satisf*) ) | 24,686 |
| S7 | S5 OR S6 | 98,584 |
| S8 | (MH "Case Management") OR (MH "Continuity of Patient Care") OR (MH "Patient Centered Care") OR (MH "Health Care Delivery, Integrated") | 73,106 |
| S9 | TI ( (integrated or "person cent*" or personcent* or "patient cent*" or patientcent* or "patient focus*" or patientfocus* or "person focus*" or personfocus* or seamless or continuity or continuum) n2 (healthcare or care or service*) ) OR AB ( (integrated or "person cent*" or personcent* or "patient cent*" or patientcent* or "patient focus*" or patientfocus* or "person focus*" or personfocus* or seamless or continuity or continuum) n2 (healthcare or care or service*) ) | 31,069 |
| S10 | S8 OR S9 | 91,353 |
| S11 | (MH "Multidisciplinary Care Team") OR (MH "Interprofessional Relations") OR (MH "Nurse-Physician Relations") | 73,403 |
| S12 | (MH "Communication Barriers") OR (MH "Collaboration") OR (MH "Communication") OR (MH "Cooperative Behavior") | 136,818 |
| S13 | S11 AND S12 | 16,003 |
| S14 | TI ( (communicat* or inform* or coordinat* or cooperat* or collaborat* or misinform* or miscommunicat*) n3 (interprofession* or "inter profession*" or interdisciplin* or "inter disciplin*" or multidisciplin* or "multi disciplin*" or "cross disciplin*" or crossdisciplin* or intersector* or "inter sector*" or "care team*") ) OR AB ( (communicat* or inform* or coordinat* or cooperat* or collaborat* or misinform* or miscommunicat*) n3 (interprofession* or "inter profession*" or interdisciplin* or "inter disciplin*" or multidisciplin* or "multi disciplin*" or "cross disciplin*" or crossdisciplin* or intersector* or "inter sector*" or "care team*") ) | 10,791 |
| S15 | S13 OR S14 | 24,157 |
| S16 | S10 OR S15 | 112,144 |
| S17 | (MH "Community Health Centers") OR (MH "Community Mental Health Services") OR (MH "Community Health Services") OR (MH "Home Health Care") OR (MH "Community Health Nursing") OR (MH "Community Mental Health Nursing") OR (MH "Parish Nursing") OR (MH "Home Nursing, Professional") OR (MH "Home Health Care") OR (MH "Home Nursing") OR (MH "Senior Centers") Or (MH "Rural Health Services") OR (MH "Rural Health Centers") OR (MH "Rural Health Nursing") OR (MH "Nursing Homes") OR (MH "Respite Care") OR (MH "Homebound Patients") | 131,201 |
| S18 | TI ( (community or municipal* or home or homes or primary or local*) N2 (service* or health* or care) ) OR AB ( (community or municipal* or home or homes or primary or local*) N2 (service* or health* or care) ) | 187,106 |
| S19 | S17 OR S18 | 277,194 |
| S20 | S7 AND S16 AND S19 | 1,666 |

**Database: The Cochrane Central Register of Controlled Trials (CENTRAL)** (Cochrane Library, Wiley)

**Date:** 28.04.2021

**Hit number:** 179

| ID | Search | Hits |
| --- | --- | --- |
| #1 | MeSH descriptor: [Aged] this term only | 210015 |
| #2 | MeSH descriptor: [Aged, 80 and over] this term only | 53642 |
| #3 | MeSH descriptor: [Frail Elderly] this term only | 730 |
| #4 | MeSH descriptor: [Health Services for the Aged] this term only | 453 |
| #5 | MeSH descriptor: [Geriatrics] this term only | 206 |
| #6 | {or #1-#5} | 210725 |
| #7 | MeSH descriptor: [Focus Groups] this term only | 630 |
| #8 | MeSH descriptor: [Interviews as Topic] this term only | 1861 |
| #9 | MeSH descriptor: [Qualitative Research] this term only | 1109 |
| #10 | MeSH descriptor: [Patient Satisfaction] this term only | 11627 |
| #11 | MeSH descriptor: [Patient Preference] this term only | 773 |
| #12 | MeSH descriptor: [Narration] this term only | 192 |
| #13 | {or #7-#12} | 15367 |
| #14 | qualitative or phenomenolog* or hermeneu* | 17320 |
| #15 | #13 OR #14 | 31029 |
| #16 | #6 AND #15 | 7291 |
| #17 | (aged or elder* or eldest or old* or geriatric* or senior* or aging or ageing) NEAR/4 (experience* or perception* or perspective* or view* or opinion* or interview* or findings or preference* or focus NEXT group* or satisf*):ti,ab,kw | 3601 |
| #18 | #16 OR #17 | 10680 |
| #19 | MeSH descriptor: [Case Management] this term only | 703 |
| #20 | MeSH descriptor: [Delivery of Health Care, Integrated] this term only | 391 |
| #21 | MeSH descriptor: [Continuity of Patient Care] this term only | 617 |
| #22 | MeSH descriptor: [Patient-Centered Care] this term only | 624 |
| #23 | {or #19-#22} | 2235 |
| #24 | (integrated or person NEXT cent* or personcent* or patient NEXT cent* or patientcent* or patient NEXT focus* or patientfocus* or person NEXT focus* or personfocus* or seamless or continuity or continuum) NEAR/3 (healthcare or care or service*):ti,ab,kw | 5274 |
| #25 | #23 OR #24 | 5891 |
| #26 | MeSH descriptor: [Patient Care Team] this term only | 1710 |
| #27 | MeSH descriptor: [Interprofessional Relations] this term only | 299 |
| #28 | MeSH descriptor: [Interdisciplinary Communication] this term only | 261 |
| #29 | MeSH descriptor: [Intersectoral Collaboration] this term only | 50 |
| #30 | MeSH descriptor: [Physician-Nurse Relations] this term only | 26 |
| #31 | {or #26-#30} | 2122 |
| #32 | MeSH descriptor: [Communication Barriers] this term only | 97 |
| #33 | MeSH descriptor: [Communication] this term only | 2278 |
| #34 | MeSH descriptor: [Interdisciplinary Communication] this term only | 261 |
| #35 | MeSH descriptor: [Cooperative Behavior] this term only | 944 |
| #36 | MeSH descriptor: [Health Information Exchange] this term only | 7 |
| #37 | MeSH descriptor: [Intersectoral Collaboration] this term only | 50 |
| #38 | {or #32-#37} | 3445 |
| #39 | #31 AND #38 | 514 |
| #40 | (communicat* or inform* or coordinat* or cooperat* or collaborat* or misinform* or miscommunicat* or mis NEXT inform* or mis NEXT communicat*) NEAR/4 (interprofession* or inter NEXT profession* or interdisciplin* or inter NEXT disciplin* or multidisciplin* or multi NEXT disciplin* or crossdisciplin* or cross NEXT disciplin* or intersector* or inter NEXT sector* or care NEXT team*):ti,ab,kw | 1120 |
| #41 | #39 OR #40 | 1319 |
| #42 | #25 OR #41 | 7038 |
| #43 | #18 AND #42 | 363 |
| #44 | MeSH descriptor: [Community Health Centers] this term only | 221 |
| #45 | MeSH descriptor: [Community Mental Health Centers] this term only | 118 |
| #46 | MeSH descriptor: [Community Health Services] this term only | 1046 |
| #47 | MeSH descriptor: [Community Health Nursing] this term only | 338 |
| #48 | MeSH descriptor: [Home Health Nursing] this term only | 7 |
| #49 | MeSH descriptor: [Parish Nursing] this term only | 1 |
| #50 | MeSH descriptor: [Community Mental Health Services] this term only | 737 |
| #51 | MeSH descriptor: [Community Pharmacy Services] this term only | 266 |
| #52 | MeSH descriptor: [Home Care Services] this term only | 1855 |
| #53 | MeSH descriptor: [Home Nursing] this term only | 280 |
| #54 | MeSH descriptor: [Senior Centers] this term only | 8 |
| #55 | MeSH descriptor: [Rural Health Services] this term only | 344 |
| #56 | MeSH descriptor: [Rural Nursing] this term only | 1 |
| #57 | MeSH descriptor: [Suburban Health Services] this term only | 6 |
| #58 | MeSH descriptor: [Nursing Homes] this term only | 1321 |
| #59 | MeSH descriptor: [Respite Care] this term only | 18 |
| #60 | MeSH descriptor: [Homebound Persons] this term only | 31 |
| #61 | MeSH descriptor: [Homes for the Aged] this term only | 644 |
| #62 | MeSH descriptor: [Assisted Living Facilities] this term only | 53 |
| #63 | {or #44-#62} | 6385 |
| #64 | (community or municipal* or home or homes or primary or local*) NEAR/3 (service* or health* or care):ti,ab,kw | 51568 |
| #65 | #63 OR #64 | 53068 |
| #66 | #43 AND #65 | 179 |

**Database:** **Web of Science** Indexes=SCI-EXPANDED, SSCI, A&HCI, ESCI Timespan=1987-2021
**Date:** 29.04.2021
**Hit number:** 319

| Set |  | Results |
| --- | --- | --- |
| 1 | TS=(((aged or elder* or eldest or old* or geriatric* or senior* or aging or ageing) NEAR/3 (experience* or perception* or perspective* or view* or opinion* or interview* or findings or preference* or qualitative or phenomenolog* or hermeneu* or "focus group*" or satisf* or narrati*) )) | 89 855 |
| 2 | TS=(((integrated or "person-cent*" or personcent* or "patient-cent*" or patientcent* or "patient-focus*" or patientfocus* or "person-focus*" or personfocus* or continuity or continuum or seamless) NEAR/2 (healthcare or care or service*) )) OR TS=(((communicat* or inform* or coordinat* or cooperat* or collaborat* or misinform* or miscommunicat*) NEAR/3 (interprofession* or "inter-profession*" or interdisciplin* or "inter-disciplin*" or multidisciplin* or "multi-disciplin*" or "cross-disciplin*" or crossdisciplin* or "care team*" or intersector* or "inter-sector*") )) | 70 801 |
| 3 | TS=(((community or municipal* or home or homes or primary or local*) NEAR/2 (service* or health* or care) )) | 303 377 |
| 4 | #3 AND #2 AND #1 | 319 |

**Database:** **Google Scholar** (via Publish or perish, Versjon 7.15.2643.7260)

**Date:**  30.04.2021

**Hit number:** 300 (Selection of the first 300 references according to Google Scholar’s relevance ranking.)

"elderly|older" "Experience|view|opinion" "Integrated care"|"person centered care"|"person centred care"|"patient centered care"|"patient centred care"|"patient focused care" "Interprofessional|multidisciplinary|interdisciplinary" "Community|municipal|local"
