# Supplementary material for: Older adults’ perceptions and experiences of interprofessional communication as part of the delivery of integrated care in the primary healthcare sector: a meta-ethnography of qualitative studies
Source: BMC Geriatr. 2024 Feb 12;24:146. doi: 10.1186/s12877-024-04745-4 (PMC10863142; doi:10.1186/s12877-024-04745-4)
Supplement: Supplementary file 2 — Additional file 2. [file 12877_2024_4745_MOESM2_ESM.docx]

Additional file 2.

| Presentation of studies included | | | | |  |
| --- | --- | --- | --- | --- | --- |
| Authors (year): reference | Aim | Participants (mean age) | Method | Analysis | Context |
| Aerts, Van Bogaert, Bastiaens and Peremans (2020): 21 | To explore the views and experiences of general practitioners, practice nurses and patients on interprofessional collaboration in general practice and to understand to what extent the nurse-doctor relationship meets their needs and expectations. | 21 patients (63.1)    19 practise nurses  7 General practitioners | Semi-structured interviews.  Pragmatic. | Thematic synthesis  (Secondary analysis) | Belgium. General practice. Primary care. |
| Borgsteede, Graafland-Riedstra, Deliens, Francke, van Eijk and Willems (2006): 22 | To explore the aspects valued by both patients and GPs in end-of-life care at home, and to reflect upon the results in the context of future developments in primary care. | 30 patients (78)    31 General practitioners | Semi-structured interviews. | Not specified | Netherlands. End-of-life care at home. Primary care. |
| Dahlke, Steil, Freund-Heritage, Colborne, Labonte and Wagg (2018): 20 | To examine older people and their families’ perceptions of their experiences of interprofessional teams. | 9 older adults (82)    13 family members | Semi-structured interviews.  Descriptive. | Content analysis | Canada. Healthcare teams. Primary care and hospital. |
| Doekhie, Starting, Buljac-Samardzic, can de Bovenkamp and Paauwe (2018): 35 | This study openly explores different perspectives of patients, informal caregivers and primary care professionals on patient involvement in primary care team interactions. | 19 older adults (81.6)    10 informal caregivers  38 primary care professionals | Semi-structured interviews.  Phenomenological. | Content analysis | Netherlands. Health care team. Primary care. |
| Eloranta, Arve and Routasalo (2008): 34 | To describe the experiences of multiprofessional collaboration in promoting personal resources among older home care clients (75+) in Finland. | 21 older adults (83.3) | Unstructured interviews.  Descriptive. | Content analysis | Finland. Home health. Primary care. |
| Lyons, Salsbury, Hondras, Jones, Andresen and Goertz (2013): 31 | To describe the preferences of older adults for LBP co-management by MDs and DCs and to identify their concerns for receiving care under such a treatment model. | 48 older adults (75.2) | Focus group interviews | Thematic content analysis | USA. Collaboration between medical doctors and Doctors of Chiropractic. Primary care. |
| Lafortune, Huson, Santi and Stolee (2015): 33 | Sought input from clients, informal caregivers, and health care providers on recommendations for system improvement | 28 clients and caregivers* (>65) | Focus group interviews.  One individual interview. | Content analysis | Canada. Community-based primary health care. |
| Abu Al Hamayel, Isenberg, Hannum, Sixon, Smith and Dy (2018): 37 | To explore older patients’ perspectives on the quality of serious illness care in primary care. | 20 older adults (70.6) | Semi-structured interviews. | Thematic analysis | USA. Serious illness. Primary care. |
| Nelson and Arnold-Powers (2001): 36 | Explore the experience and illuminate the needs of frail older people who work with a community case manager in a managed care setting. | 11 older adults (age 67–89) | Semi-structured interviews.  Descriptive | Not specified | USA. Case management. Primary care. |
| Oosterveld-Vlug, Custers, Hofstede, Donker, Rijken, Korevaar et al. (2019): 32 | Exploring what care users find important aspects of good-quality palliative care and relating this to their actual experiences | 13 patients (age 58-86)  14 relatives | Semi-structured interviews. | Thematic analysis | Netherlands. Palliative care at home. Primary care. |
| Wells, Salsbury, Nightingale, Derby, Lawrence and Goertz (2020): 30 | To explore older adults’ perceptions of the healthcare processes, including doctor–patient relationships and health communication, that they experienced during a clinical trial of interprofessional care for LBP. | 115 older adults (72.9) | Semi-structured interviews.  Descriptive. | Thematic analysis  (Secondary analysis) | USA. Interprofessional care in an LBP treatment intervention. Primary care. |
| *Number of clients and caregivers were not specified | | | | | |
